# [Corrigendum] Inhibition of Rac1 reverses enzalutamide resistance in castration-resistant prostate cancer

**DOI:** 10.3892/ol.2026.15523

**Published:** 2026-03-11

**Authors:** Xiaoliang Chen, Lili Yin, Gan Qiao, Yanhua Li, Baoyuan Li, Yunfeng Bai, Feng Feng

Oncol Lett 20: 2997–3005, 2020; DOI: 10.3892/ol.2020.11823

Following the publication of the above article, an interested reader drew to the authors' attention that, for the Transwell migration assay experiments shown in Fig. 3A on p. 3001, the sh-Ctl/DMSO and sh-Ct/Enza data panels appeared to contain an overlapping section of data, such that these data panels were apparently derived from the same original source where the results of different experiments were intended to have been portrayed.

The authors were able to consult their original data, and realized that this figure had inadvertently been assembled incorrectly. The revised version of Fig. 3, now showing the correct data for the sh-Ct/Enza panel, is shown on the next page. The authors regret that this error occurred in the original published version of this figure, although this did not grossly affect the results or the conclusions reported in this article. All the authors agree with the publication of this Corrigendum, and thank the Editor of *Oncology Letters* for allowing them the opportunity to publish this; furthermore, they apologize to the readership for any inconvenience caused.

## Figures and Tables

**Figure 3.**
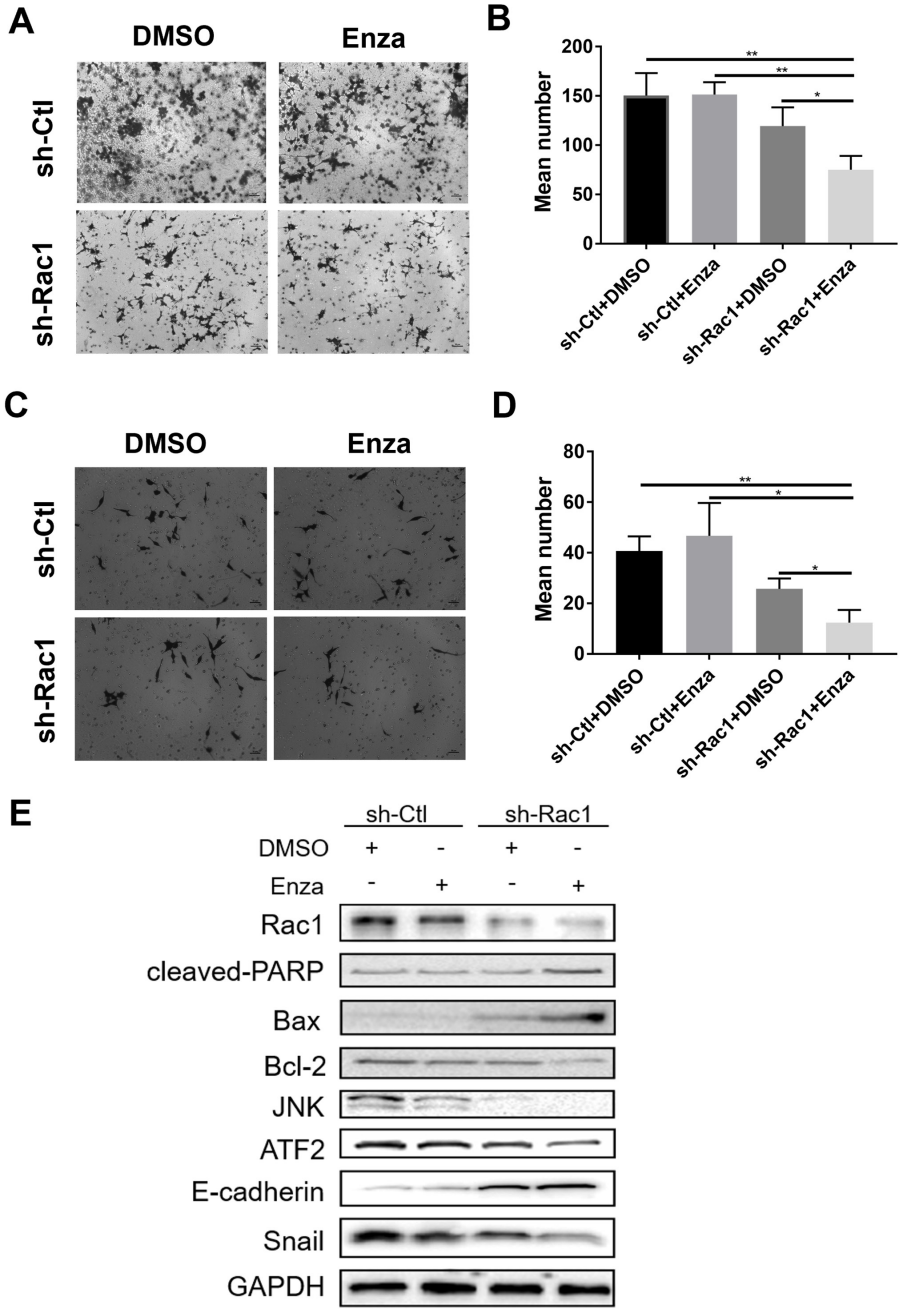
Knockdown of Rac1 attenuates migration and invasion of Enza-resistant PCa cells. (A-D) MR49F cells were depleted of Rac1 using an shRNA lentivirus and then treated with or without 10 µM Enza followed by analysis of cell (A and B) migration and (C and D) invasion using Transwell assay at ×100 magnification. (B and D) Quantification of the number of (B) migration and the invasion (D) cells. (E) Western blotting was used to assess the mechanism of the enzalutamide-induced inhibitory effect on apoptosis and EMT. Data are presented as the mean ± SD of three independent experiments. *P<0.05, **P<0.01. Rac1, Ras-related C3 botulinum toxin substrate 1; shRNA, short hairpin RNA; Ctl, control; Enza, enzalutamide; ATF2, activated transcription factor 2.

